# The Effect of Immediate Obturator Reconstruction after Radical Maxillary Resections on Speech and other Functions

**DOI:** 10.3390/dj6030022

**Published:** 2018-06-21

**Authors:** Mehmet Dalkiz, Ahmed Suat Dalkiz

**Affiliations:** 1 Private Practice 1, Boulevard Sylvain Dupuis 235-10, 1070 Brussels, Belgium; 2 Private Practice 2, Boulevard Sylvain Dupuis 235-10, 1070 Brussels, Belgium; asdalkiz@hotmail.com

**Keywords:** immediate obturator, immediate prosthodontic, reconstruction, maxillary tumor resections

## Abstract

Objective: Maxillectomy often results in a high level of morbidity with significant psychological and functional implications for patients. The aims of the present study were to assess the effectiveness of the maxillary obturator as a speech rehabilitation aid, to examine the influence of dentition on speech intelligibility, to restore patients’ regular daily activity as soon as possible, and to maintain patients’ psychological well-being throughout the treatment. Patients and Methods: Forty-one palatomaxillary immediate obturator and definitive reconstruction patient treatments were reviewed at a clinic (Ankara, Turkey). Patients aged between 20 and 73 years with surgically acquired partial maxillary defects were included in this study. All patients were rehabilitated with immediate and definitive obturators. The patients were given immediate surgical obturators which were adjusted to the defect area with a tissue conditioner. By employing this procedure and relining with the tissue conditioner weekly, immediate obturators were used in the interim stage of the treatment. As interim obturators, prostheses were used for two to three months until healing and resorption were found satisfactory, after which the definitive obturators were fabricated. Results: The speech intelligibility test (SIT) was employed for the evaluation of the speech ability. Significant improvements were found in the mean speech intelligibility test score (SITS), from 0.02% in patients without prosthetic obturation to 94.10% in patients with immediate obturation on the second day, 95.60% in patients with immediate obturation on the 20th day, and 95.97% in patients with definitive obturation.

## 1. Introduction

Maxillofacial deformities can be congenital, acquired, and developmental [1,2,3,4,5]. An obturator is a maxillofacial prosthesis that is used to close and maintain the integrity of the oral and nasal compartments that are altered because of a congenital, acquired, or developmental disease. Often, reconstructive surgery alone is not enough to restore the defects, especially when a defect is relatively large; hence, prosthetic reconstruction must be employed [6,7].

Head and neck defects can be restored with the help of maxillofacial prosthetic rehabilitation to near-normal function and aesthetics. The prosthetic rehabilitation for maxillectomy patients aims at the separation of the oral and nasal cavities to allow adequate deglutition and articulation in order to restore the mid facial contour and to provide acceptable results [7,8].

The management of most head and neck neoplasms usually involves radical surgical resection, chemotherapy, and radiotherapy [9,10,11,12]. As organ preservation has become an important goal of oncologic treatment, efforts are being made to preserve phonation and deglutition without compromising therapy efficacy [7,13]. Surgical reconstruction may be the treatment choice to restore the integrity or continuity of the affected structure after the resection; however, it may not always be possible [14]. In such cases, prosthetic rehabilitation of the defects provides the option of regaining functional integrity and esthetics while improving the patient’s quality of life during or after life-saving treatments [15,16]. The main objectives of maxillofacial prosthetic rehabilitation are the restoration of orofacial functions such as obturation, deformation, control of secretions, chewing and phonetics, and at the same time the correction of the changing aesthetic of missing orofacial structures [16]. The initial planning for patients of head and neck neoplasms should include an evaluation by a prosthodontist. Even if surgical reconstruction is planned, dental prosthesis may be considered a part of the definitive rehabilitation. The expertise of a dental specialist is necessary to address subtle dentoalveolar concerns that may impact decision-making, such as plane of occlusion, articulation of teeth, tooth mobility, pre-existing temporomandibular joint dysfunctions, orodental parafunctional habits (e.g., nocturnal grinding or clenching of the teeth), pre-existing benign conditions, anatomic variations, and postoperative orodental care.

Prosthodontic management can be divided into three phases:

1. Pre-operative construction of a prosthesis and insertion of it at the time of operation.
2. Post-operative modification of the prosthesis during the recovery period.
3. Construction of a definite prosthesis employing all the established principles of prosthodontics, when the healing is complete, and prognosis is not questionable [7].

The surgical obturator is the first prosthesis that is inserted at the time of surgery and helps the patient prevent oral contaminations. Interim obturators are fabricated on a postoperative cast, so they are more accurate than the immediate surgical obturator. The interim prosthesis is periodically readapted and, if required, it is relined to adjust the tissue changes occurring during the healing period of the defect. This improves patient function and comfort. A definitive obturator is started approximately three to four months after surgery, when healing is complete. A custom tray is required for the definitive impression because proper extension and adequate contour of the tray are essential for a successful impression [17,18,19,20,21,22,23].

The aims of the present study were to examine the influence of maxillary immediate obturators on speech intelligibility and to evaluate the treatment of mastication and swallowing. In order to restore oral cavity function, the patients’ regular daily activity should be recovered as soon as possible, which also helps to maintain the patients’ psychological well-being throughout the treatment.

## 2. Patients and Methods

**Figure 1:**
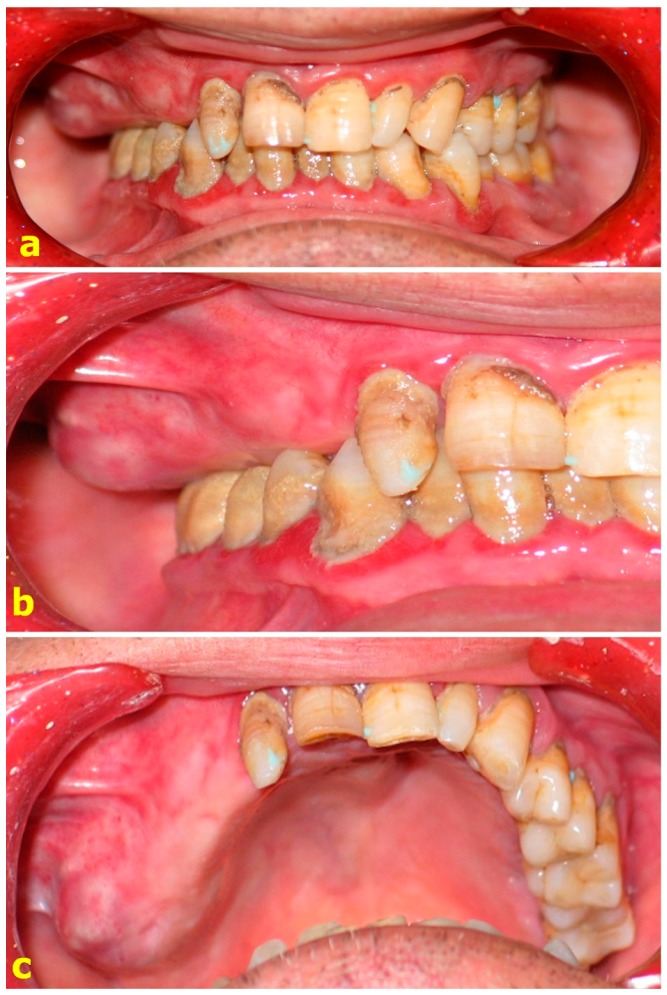
Clinical aspect of a lesion. (**a**,**b**) Pre-operative intraoral view of the lesion. (**c**) The lesion is in the edentate region to the right of the maxilla.

**Figure 2:**
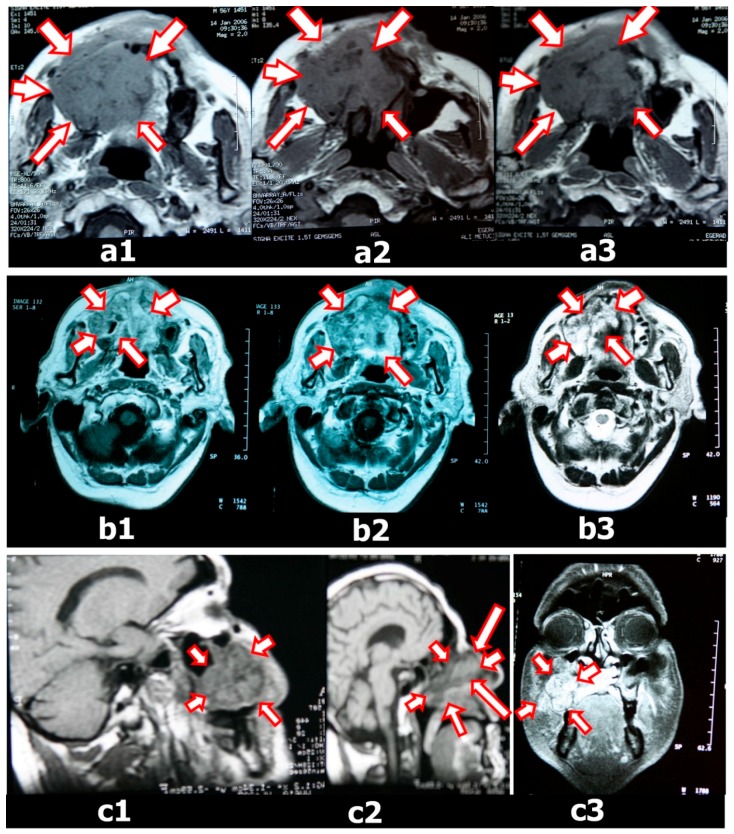
Axial and Coronal MRI weighted images showing a lesion of the maxillofacial region. (**a1–3**) MRI images of a palatomaxillar tumor on the horizontal plane before chemotherapy and radiotherapy (white arrows with red border); (**b1–3**) MRI images of the palatomaxillar tumor on the horizontal plane after chemotherapy and radiotherapy (white arrows with red border); (**c**) MRI images of the palatomaxillar tumor on the sagittal (**c1**,**2**) and frontal (**c3**) plane after chemotherapy and radiotherapy (white arrows with red border).

**Figure 3:**
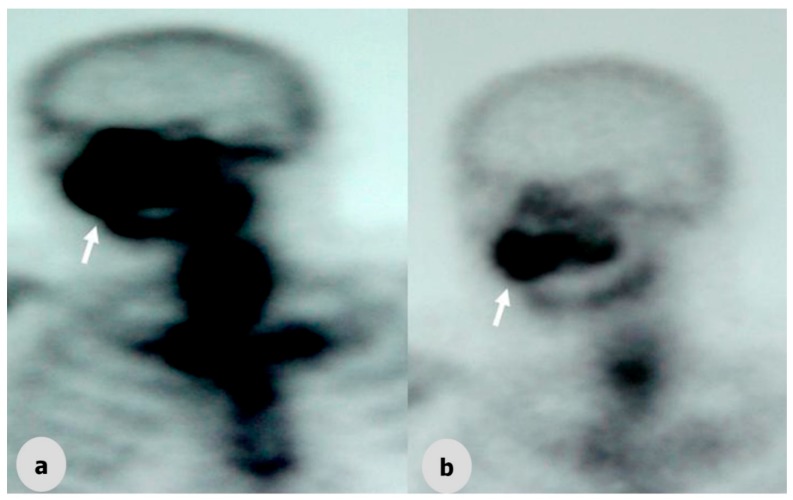
Scintigraphy images of a tumor (arrow) of the maxillofacial region before (**a**) and after (**b**) radiotherapy.

In this study, forty-one patients aged between 20 and 73 years with surgically acquired partial maxillary defects were included. Ten of these patients were edentulous, twenty were partially edentulous, and eleven were dentulous before the surgery (Figure 1, Figure 2 and Figure 3). The consents of the patients and the ethical committee were obtained.

Pre-surgical dental and oral exams were done to determine the number, location, and integrity of the remaining teeth, the status of the dentition in the opposing arch, and the size and arch form of the maxilla. Diagnostic casts of both arches were made. General anesthesia was used for maxillectomy. Either orotracheal or nasotracheal intubation were selected depending on the surgical approach. The patients’ eyes were protected carefully. Preoperative antibiotics were prescribed and continued 10 days postoperatively. The choice of the surgical approach was determined by the location, size, type, and aggressiveness of the tumor, the extent of the planned resection, and the preferences of the patient, the surgical team, and the prosthodontist. Lesions were usually accessed via a facial approach (the extraoral approach). The incisions were outlined at the margin around the tumor, depending on the histopathology observed in the biopsy. Once all the tumor excision were complete, bleeding was controlled with bipolar cautery and figure-of-eight suture or legating clips. Dental implants were not implanted in any patient. Two obturators were delivered for the whole period of treatment and rehabilitation. Obturators included three phases: immediate (surgical) obturator (placed at the time of surgery); an interim surgical obturator (fabricated to aid in the healing of tissues during the recovery period, 3–4 weeks after surgery); a definitive obturator, after three months.

The immediate or surgical obturation refers to the immediate coverage of a palatal defect with an obturator. Using obturators minimizes wound contamination and enables the patient to speak, swallow, and eat effectively immediately after surgery.

Before tumor resection, a dental impression was made using irreversible hydrocolloid impression material for fabrication of the immediate surgical obturator. Dental casts were fabricated with dental stone. The surgical limits were determined on casts with 3D tomography and MRI. The dental cast was cut from these boundaries. On the maxillary diagnostic cast, the surgical margins were plotted approximately whilst consulting with the surgical team because complete coverage of the surgical site with the obturator is crucial. Immediate obturators were prepared on these casts (Figure 3).

**Figure 4:**
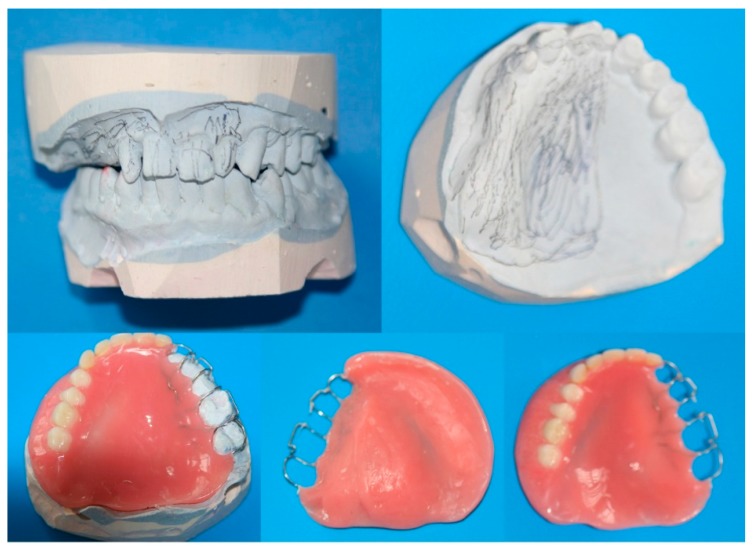
The surgical team plotted the surgical margins on the dental cast and the fabricated prosthesis (immediate surgical obturator).

**Figure 5:**
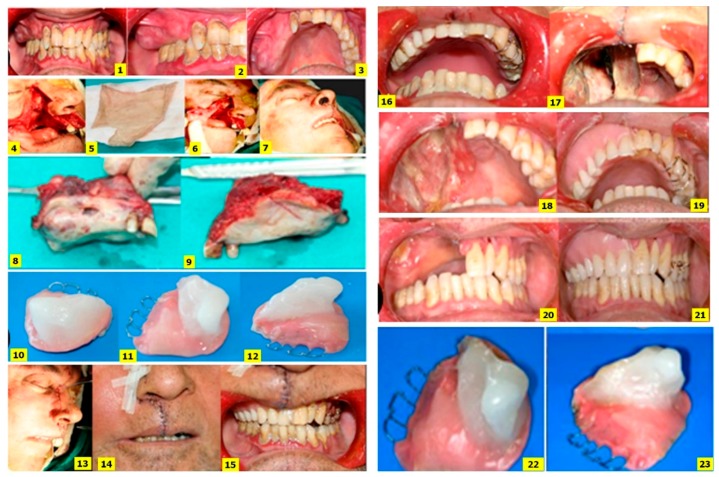
Clinical images show the affected area before surgery, during surgical intervention, and after surgical intervention with immediate obturator application. (**1**–**3**) The affected area before the surgery; (**4**) the incision line and surgical area; (**5**) split thickness skin graft; (**6**) placement of the graft (split thickness skin graft) onto the recipient site (surgical area), and wound covered with a split-thickness skin graft; (**7**) final appearance of the patient following the adjustment of the obturator; (**8**,**9**) resected tumor specimen; (**10**–**12**) adaptation of the immediate obturator with the tissue conditioner; (**13**) end of the surgical intervention; (**14**) post-operative day one; (**15**) post-operative day three; (**16**) occlusal view of the immediate obturator on the third day; (**17**) surgical defect on day three; (**18**,**19**) view of the postoperative surgical defect and immediate obturator on day 10; (**20**) acquired defect and (**21**) immediate obturator with tissue conditioner one month after surgery; (**22**,**23**) view of the immediate obturator with tissue conditioner one month after surgery.

The surgical obturators were then fabricated as dentures using conventional techniques (Figure 4) and inserted at the time of surgery by adjusting to the contours of the defect area using a tissue conditioner. After ten days, these obturators were relined with the tissue conditioner, and the relining procedure was repeated weekly, making the immediate obturator suitable for use as an interim prosthesis (Figure 5).

**Figure 6:**
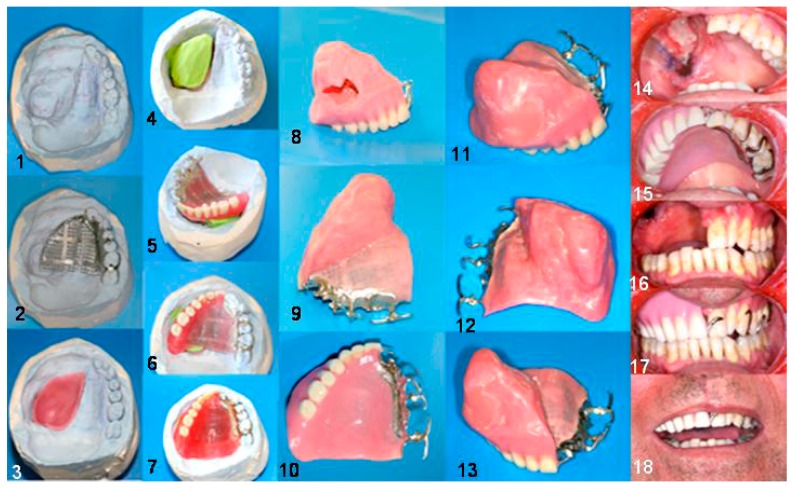
Fabrication of the definitive obturator. (**1**) Cast of the maxilla; (**2**) metal frame on the cast; (**3**–**6**) preparation of the hollow bulb; (**7**) modulation finished for the flusking procedure; (**8**) view of the bulb after removing the polysiloxan impression material; (**9**–**13**) definitive obturator; (**14**,**16**) defect area; (**15**,**17**,**18**) obturator inserted.

These patients were scheduled for the first two to three months immediately. After this, the interim obturator was placed, and then, six months later, the definitive obturator was placed. The interim stage of the treatment was two to three months long until healing and resorption were found to be satisfactory. In all cases, the definitive obturators were fabricated, inserted, and checked for fluid leakage, speech intelligibility, and aesthetics. In the absence of fluid leakage, further relining was unnecessary. Frequent recall visits were planned for the first three months and were gradually reduced to once every three months (Figure 6). The patients often complained about eating and drinking difficulties and issues during speech.

The SITS (speech intelligibility test score) described by Plank et al. [1] and Wheeler et al. [2] was employed in this study. Each subject’s reading of a text of 20 words was tape-recorded on five occasions:

1. Before the surgery.
2. Second post-operative day with and without the obturator.
3. Twentieth post-operative day without the obturator.
4. After the insertion of the interim obturator (the immediate obturator was converted to an interim obturator by relining with the tissue conditioner) on the twentieth post-operative day.
5. Following delivery of the definitive obturator, two to three months post-operation.

Recordings were made with the subjects seated comfortably in a quiet room facing a tape recorder placed approximately 15 cm from each speaker’s lips. Each of the speech recordings outlined above was presented to a group of 10 Turkish listeners through earphones one after the other. A group of 10 listeners evaluated the speech recorded without an obturator, while a different group of 10 listeners evaluated the speech recorded with the interim obturator, and another group of 10 listeners evaluated the speech recorded with the definitive obturators. The listeners were untrained (listeners with no prior experience of speech assessment), with no prior exposure to the message being evaluated, and not familiar with the surgical procedures that had been performed. 

The listeners were instructed to write down what each patient said. The number of words correctly understood and written by the listeners was recorded. Each patient was given an intelligibility score recorded by the listener. An intelligibility score represents the percentage of items (in these cases, words) correctly identified or understood. The response of each listener was evaluated by counting the number of words spoken and intended by the speaker and those correctly understood by each listener and then finding the percentage. The understandability score represents the percentage of the items that are correctly defined or understood (in this case, words). The correct word numbers and percentages are 0 (0%), 1 (5%), 2 (10%), 3 (15%), 4 (20%), 5 (25%), 6 (30%), 7 (35%), 8 (40%), 9 (45%), 9 (50%), 10 (55%),11 (60%), 12 (65%), 13 (70%), 14 (75%), 15 (80%), 16 (85%), 17 (90%), 18 (95%), and 20 (100%). After the evaluation of the responses of the listeners, an average score which represents the SITS of wach patient was calculated (Table 1).

The data was analyzed using the Statistical Package for the Social Sciences (SPSS) version 11 (Inc. Standard version 2001). The analysis included frequency, calculation of mean values, and standard deviations. The differences between the means were tested using a grouping variable and the Kruskal-Wallis Test (Table 2 and Table 3).

## 3. Results

For this study, 41 patients with surgically acquired maxillary defects were divided into three groups: edentulous, partially edentulous, and dentulous. SIT scores of the groups were recorded before the surgical operation, on the second and twentieth days of the immediate obturation, and at the definitive stage of the treatment. The lowest percentage mean SI score of 0.02% was recorded without prosthetic obturation, while this mean value increased to 94.10% on the second day and to 95.60% on the twentieth day of immediate obturation. The highest percentage mean SITS score recorded was 95.97% following definitive obturation (Table 1).

The Kruskal-Wallis test and grouping variable procedure were used to determine whether there were significant differences in the mean SITS of the study groups and in the various stages of treatment (Table 2).

Separate comparisons were made both between the three groups and between the treatment stages for each group. According to the results given in the Table 2, no significant differences could be found comparing the groups to each other before the surgery (*χ*^2^ = 0.000; *p* = 1.000), on the second day after obturator removal (*χ*^2^ = 1.050; *p* = 0.592), and on the second day with the obturator inserted (χ^2^ = 0.842; *p* = 0.656 and 20th days of post-operative stage, obturator removed (*χ*^2^ = 1.051; *p* = 0.593), 20th days of post-operative stage with the obturator inserted (*χ*^2^= 3.679; *p* = 0.159), and at the definitive stage of the treatment with the definitive obturator inserted (*χ*^2^ = 3.438; *p* = 0.179). As the intelligibility rates of the **E** (edentualism) group were studied, no significant differences were shown between the recorded scores (*χ*^2^ = 1.684; *p* = 0.431). As the intelligibility rates of the **PE** (partial edentualism) group were studied, no significant differences were shown between the recorded scores (*χ*^2^ = 11.793; *p* = 0.003). As the intelligibility rates of the **D** (dentualism) group were studied, no significant differences were shown between the recorded scores (*χ*^2^ = 1.727; *p* = 0.422). As the intelligibility rates of the post-operative group without the obturator on the second day and of the post-operative group with the obturator on the 20th day were studied, significant differences were shown between the recorded scores (*z* = 2.414; *p* = 0.016). As the intelligibility rates of the post-operative group without the obturator on the second day and of the post-operative group with the definitive obturator were studied, significant differences were shown between the recorded scores (*z* = 2.565; *p* = 0.010). As the intelligibility rates of the post-operative with the obturator on the 20th day and of the post-operative group with the definitive obturator were studied, no significant differences were shown between the recorded scores (*z* = 0.477; *p* = 0.655). As the intelligibility rates of all groups with the obturator and of all groups without the obturator groups were studied, significant differences were shown between the recorded scores.

As the intelligibility rates of each group were studied, significant differences were shown between the recorded scores:

- At the pre-operative stage and on the second day of the postoperative stage without the obturators (<0.001).
- On the second day of the post-operative stage without the obturator and with the obturator inserted on the same day (<0.001), on the 20th day (<0.001), and at the definitive stage with the definitive obturator (<0.001) (Table 3).

The review of these data reveals that communication performance and the presence of the teeth are not correlated. Also, the results indicate that the prosthodontic intervention was highly successful for the rehabilitation of the speech of the patients with maxillary defects.

## 4. Discussion

Surgical resection is an established and common method for the treatment of maxillofacial cancer. Acquired surgical defects of hard and soft palates interfere with the speech pattern and mechanism of deglutition. Patients with maxillofacial defects labor under handicaps that cannot be fully appreciated by normal people. Ideally, any anatomic defect should be surgically reconstructed. Often, reconstructive surgery alone is not enough to restore the defects, especially when the defect is large, so prosthetic reconstruction must be employed. Although surgery is a common approach to the treatment of maxillofacial pathologies, it may not be possible or practical for numerous clinical situations. In these instances, prosthetic treatment combined with mastication and speech therapy may be the treatment of choice. Head and neck defects can be restored with the help of maxillofacial prosthetic rehabilitation to near-normal function and esthetics. The prosthetic rehabilitation for maxillectomy patients aims at the separation of the oral and nasal cavities to allow adequate deglutition and articulation, to restore the mid-facial contour, and to provide acceptable results. It is important to familiarize the patient with the functional and cosmetic expectations and limitations of the maxillofacial prosthesis.

The obturators are easy to produce and offer functional restoration without the need for additional surgery. In previous studies, the immediate obturators had been fabricated 10 days after surgical intervention. In this study, however, the immediate obturator was pre-surgically fabricated and adjusted to fit the defect at the time of surgery, using tissue conditioners. With this adjustment, the necessity to use surgical packs and nasogastric tube is eliminated. All patients who used immediate obturators had speech, comfort, convenience, and comfort advantages with social interactions and could also chew because of the presence of teeth included in the design, contrary to the ones who did not have obturators during the healing process.

The first target of the immediate obturators is to provide support for the grafts and surgical dressing placed in the defect. The obturator separates the maxillary surgical site from the oral cavity. This helps in speaking, mastication, and swallowing more normally after surgical intervention. The obturator restores the patient’s lost maxillofacial contours, oral structures, and esthetic. This allows the patient to live in a psychosocial environment [24].

Effective communication between the treatment teams is needed for successful treatment and rehabilitation. This is important to determine the surgical margin and to design the immediate obturators. The major goal of therapy is not only to eradicate the disease, but also to give patients a reasonably normal life. The team concept is to ensure the patient’s early and successful rehabilitation [25,26]. Immediate obturators reduce both post-operative morbidity and length of hospitalization. The main purpose of rehabilitation is to restore the lost functions and esthetics. Generally, the first and most important problem of the patients is probably the speaking disorder following surgical intervention

An immediate obturator prosthesis is required for the restoration of speech, deglutition, and improvement in esthetics after maxillectomy. Immediate obturators are fabricated on a pre-operative cast and they are periodically readapted to adjust the tissue changes during the healing period of the defect. Prosthetic reconstruction of patients with maxillofacial defects can be divided into three stages (immediate/surgical obturator, transient/temporary obturator, and definitive obturator). The immediate obturator can be used as a temporary/interim obturator. Obturators separate the mouth and the nasal cavity, restoring swallowing and speech functions. In addition, the obturator reduces the psychological effect of surgery and simplifies the rehabilitation procedures.

Obturators are the first choice and most effective treatment for patients with maxillectomy. For increase speech intelligibility and effective chewing, the obturator must tightly close to prevent air and fluid passage between the mouth and nose. The obturator should be prepared as a hollow bulb to increase and relieve retention and stability. This can sometimes cause patient dissatisfaction. The hollow maxillary obturator prosthesis can reduce the weight of the prosthesis by 7–33%, depending upon the size of the maxillary defect [27,28,29]. The challenge in rehabilitating a maxillectomy patient is to obtain adequate retention, stability, and support. A hollow bulb obturator allows for the fabrication of a lightweight prosthesis, along with adequate extensions within the prostheses, making it tolerable for the patient. The definitive closed hollow obturator helps to achieve the primary objective of restoring the functions of mastication, speech, and aesthetics. 

In this study, it was observed that immediate obturator reconstruction of the maxillectomy defect has a significant relationship with the articulation of speech and other oral functions. The remaining maxillary teeth and supported tissues influence the respiration, mastication, swallowing, and articulation of speech. The size of the nasal extension within the defect can differ depending on the location, size, and shape of the defect, the defect surfaces, and the functional requirements for prosthesis retention and stabilization [11,30]. In large defects with loss of soft and hard tissue support, the obturators are extended to engage the surgical defect, so they have a large size and are heavy in weight; large obturators are less effective in the oral functions. In this context, Adisman [30], reported that obturators are suitable to cover the hard palate defect with minimal undercuts and create a seal. Aramany and Drane [31] discovered that the nasal extensions of hollow obturators tend to improve voice quality in patients with large palatal defects, while minor defects showed that the nasal extensions had not much effect on the speech quality [32]. Suha et al. [33] reported that the nasal cavity should be positioned firmly in the obturator defect area to prevent air, fluid, and food leakage; however, it has been declared that an obturator placed tightly in the defect with a tight closure is affected by the soft tissue around the prosthesis and the mouth functions [34]. In large defects which lack palatal support, the obturator is mostly extended vertically and horizontally to engage the surgical defect. Therefore, it expands its size and weight. The remaining structures are subjected to continuous stresses from such large, heavy obturators and reduce a patient’s function and comfort.

A hollow bulb obturator with a hollowed-out denture considerably reduces the weight of the prosthesis, increases retention and thus improve the physiologic functions, such as deglutition, improves patient comfort and efficiency, decreases the pressure on the surrounding tissues, results in a good regeneration of tissues, reduces the chances of excessive atrophy and physiologic changes in the muscle balance, and improves the self-confidence of the patient. In this study, permanent prostheses were made in the form of a hollow bulb obturator to reduce the weight of obturators. The technique reduced the weight by approximately 20%, which helped achieve better retention, stability, and support of the obturator that was part of a maxillary obturator. The patients, after wearing the prosthesis, had better comfort, function, speech, and appearance. Speech intelligibility is enhanced by the use of, firstly, an immediate obturator and, secondly, a definitive hollow bulb obturator. Immediate obturator intervention should be considered as an integral component of soft palate resection, resulting in an excellent restoration of velopharyngeal insufficiency, thus providing the patients with an acceptable and functional speech outcome.

Bohle et al. [35] demonstrated that, as the size of a palatal defect increases, the intelligibility of speaking decreases. Sullivan et al. [23] observed that the defect can change speech intelligibility depending on its position in the hard and soft palate. Moreover, maxillectomies, particularly the largest ones, restrict the contact between the tongue and the palate, impairing speech intelligibility. 

The speech intelligibility scores in individuals with normal speech between the ages of 40 and 75 years were found to be 90% and higher [23,36,37,38,39,40,41,42]. Rieger et al. [22] found that the scores ranged between 93% and 98% in their study. In this current study, the preoperative intelligibility scores were 94.10–95.60%. Thus, the preoperative measurements within the current investigation represent a valid control condition, and the presence of the maxillary lesion appears to be of no consequence to the preoperative speech measurements collected herein. The results of this study indicate that speech can be functionally restored to a preoperative level with an immediate obturator. This investigation shows no significant differences between speech measured at the preoperative time and that measured after surgery when the patient was wearing an immediate or a definitive obturator. In a study done by Umino et al. [37], the lowest mean SI (speech intelligibility) score of 35.7% was recorded without obturation. Also, the lowest reported mean SI scores of 61% by Sullivan et al. [23] and 59.8% by Arigbede et al. [38] were for patients without obturation. In our study, the lowest mean intelligibility score of 0.02% was recorded before obturation.

Fenn et al. [39] thought that, after the maxillary resection, the low mean SITS of a patient without obturator depended on the ora-antral communication. They stated that the oro-antral communication abolished or impeded the patient’s ability to speak, thus forcing the air to proceed through the mouth to pronounce all vowels and most consonant sounds. Following obturation, however, the impairment was eliminated, and the SITS improved. The mean score of 0.02% recorded without obturation in this study increased to 94.1% on the second day of immediate obturation and to 95.6% on the twentieth day. This is similar to the results reported by Umino et al. [37]. This improvement was due to the sealing of the oro-antral communication, the ability of the tongue to articulate sounds by the seal produced by the tip of the tongue, and the immediate obturator [18]. Arigbede et al. [38] recorded the highest mean intelligibility score of 94.7% following definitive obturation. The highest mean intelligibility score recorded in this study was 95.97% following definitive obturation. This value is comparable to the mean score of 94.7% recorded by Plank et al. [1] Arigbede et al. [38] reported that this improvement may be a result of the addition of teeth to the obturator, a proper seal produced by the definitive obturator after complete healing, and the resonation of sounds produced by the hollow bulb design of the obturator [23]. The presence of the teeth on the immediate obturator design used in our study gave us this advantage from the beginning of the immediate stage.

Before maxillectomy, all patients had speech intelligibility scores that were about 100% correct, similar to those of healthy individuals, indicating that the presence of tumors did not impair speech intelligibility. Without an immediate obturator prosthesis, on the second day after surgery, all patients achieved scores that were about 0.02%. All patients exhibited a significant reduction in speech intelligibility scores without prosthesis following surgery. In all patients with immediate obturator prosthesis, scores similar to healthy subjects were obtained at 94.10% on the second postoperative day and at 95.60% on the postoperative day 20. Comparison of assessments of speech intelligibility in patients with and without prosthesis conducted by Carvalho-Teles et al. [43] showed that under the condition without prosthesis, 15 patients (65.2%) presented poor levels of speech intelligibility, while in the remaining eight patients (34.8%), intelligibility ranged from mild to normal. Speech disorders following maxillectomy are related to oro-nasal opening, loss of orofacial tissues, and incorrect palatal contact of the tongue [18]. Several researchers have reported that obturators increase speech intelligibility [1,31,44,45,46]. However, speech disorders after the prosthetic restoration of maxillary resections arise because of insufficient retention, stability, and oro-nasal sealing by the obturators [45]. In the former category, a prosthetic obturator cannot provide the seal necessary to ensure effective oro-nasal separation [47]. In the latter category, the velopharyngeal function is adversely affected by destroying the attachment for the palatal musculature, by simultaneous denervation of the palatal muscles, or by the relative shrinkage and immobilization of the soft palate through scar contracture [31,42,43,44,45,46,47,48,49].

The relationship between speech intelligibility scores and oro-nasal separation, velopharyngeal function, retention, and stability of prostheses following the placement of maxillary obturator prostheses is shown in Table 1 and Table 2. The results indicate that the satisfactory improvement in speech was attributable to a stable prosthesis. Yoshida et al. [46] and Curtis and Beumer [18] suggested that the palatal lift extension of the obturator can help velopharyngeal closure, when the scar contracture or innervation disorder result in palatopharyngeal insufficiency. In this study, the use of obturators led to a normal velopharyngeal function, resulting in the development of speech intelligibility. The main scale of communication is speech intelligibility, because comprehensible speech is one of the most important social communication tools. In this current study, in all patients, all assessments of SITS in the presence of obturators proved improvement in speech intelligibility, in agreement with results reported in the literature [18,22,23,37,49,50].

Lawson [41] reported that, in order to pronounce certain consonants clearly, a lateral seal produced by the sides of the dorsum of the tongue making contact with the upper posterior teeth is necessary. Similarly, Fenn et al [39] and Kornblith et al. [50] stated that, to pronounce consonants like F, V (labiodental sound), and Th (dental sound), the presence of the anterior teeth is required. Therefore, without tongue, teeth, and the partial or complete absence of maxilla, difficulty can be experienced in making the required contacts with the tongue, teeth, and palatal surface, required to produce various speech sounds. The addition of artificial teeth to an immediate obturator, then, caused less difficulty in pronouncing words and less changed the speech intelligibility after obturator rehabilitation. In these studies, the small variations observed in the average SITS cannot be due to the fact that the studies were conducted with different numbers of subjects by the researchers. Plank et al. [1] included 10 patients and Sullivan et al. [23] included 34 patients. Our study was performed on 41 patients with maxillectomy.

In this study, a comparison of two assessments of speech intelligibility has shown that, under the condition without prosthesis, all patients (0.02%) presented poor levels of speech intelligibility, while, under the condition with prosthesis, all patients’ (94.10%) speech intelligibility ranged from an acceptable level to normal. Speech intelligibility and other oral functions affect the size of the maxillary defect, prosthesis stability, prosthesis weight, radiotherapy, and the time without prosthesis after a surgical intervention. According to Carvalho-Teles et al. [43] and Pegoraro-Krook [51], speech intelligibility has no relationship with SITS and before- or after-surgery speech therapy. Without the need for speech therapy, the accuracy of speech clarity has been associated with normal speech abilities before surgical intervention and palatal obturation. In the current study, all patients did not undergo any speech therapy, and the listeners were untrained in speech intelligibility. However, we can say that speech intelligibility can benefit from speech therapy for patients who do not improve with the obturator. 

Hypernasal speech is a disorder that causes abnormal resonance in the voice due to increased airflow through the nose during speech. Hypernasality reduces the quality of life because of its impact on speech intelligibility. According to Yoshida et al. [46], mild hypernasality can cause only small speech distortion, and severe hypernasality leads to a serious reduction in speech intelligibility and prevents an individual's oral and social communication. Bohle et al. [35] observed that SITS increased with the intelligibility of words and sentences. Speech intelligibility was reduced when hypernasality increased. In the present study, the speech intelligibility results have demonstrated a reduction in hypernasality with the use of the prosthesis in the patients (94.10%, 95.60%, and 95.97%), in agreement with some studies in the literature [23,35,42,50,51,52].

There was no significant correlation between variables analyzed for speech intelligibility and prosthesis variables (*p* = 0.044, *p* = 0.012 and *p* = 0.474) in the groups with the obturator, while significant connection was observed between speech intelligibility and word prononciation in the groups with and without the obturator on speech resonance (Table 2 and Table 3). These results show that obturators lead to decreased hypernasality and increased speech intelligibility. Studies have reported a significant increase in the percentage of speech intelligibility associated with a reduction in hypernasatality after prosthetic rehabilitation with an obturator [22,23,35,46,48,50,51]. These results are similar to the findings of the current study.

There were significant differences in the mean SITS scores across the three stages of prosthetic treatment. There was also a significant difference between the mean SITS score recorded without obturation and following immediate obturation, and no significant difference was found between definitive obturation and immediate obturation. These findings were not similar to the results recorded by Umino et al. [37] and Sullivan et al. [23]. This shows that the two types of obturators, namely, immediate and definitive, are indeed beneficial to the patients.

## 5. Conclusions

Oral cavity is a functionally important area essential for speech, mastication, and swallowing. Surgical resections for maxillofacial cancers are often extensive, involving complex reconstructive procedures leading to the impairment of speech, mastication, and swallowing. Despite improvements in the reconstructive armamentarium, including microvascular free flaps and sensate flaps, the magnitude of these functional impairments still persists, which in turn adversely affects the psychosocial wellbeing of the patients.

An immediate obturator is required for the restoration of eating and drinking, for the correction of speech and deglutition, and for the improvement in aesthetics after maxillectomy. Obturators are fabricated on a pre-operative and post-operative cast and they are periodically readapted to adjust the tissue changes during the healing period of the defects. Immediate obturators provide an improved quality of life and functional advantage during the healing period.

In this study, patients with a maxillectomy defect who had velopharyngeal insufficiency were successfully treated by the obturators. We therefore emphasize that properly fabricated obturators can help restore the anatomy and function of the lost tissues and go a long way in the rehabilitation and in improving the quality of life of patients.

The performance of obturators can be limited by factors such as radiotherapy, chemotherapy, and degree of surgery. An interdisciplinary treatment team is required in order to increase the success of treatment and the quality of life of the patients, while the obturator prosthesis contributes to improved speech intelligibility, good maxillofacial appearance, improved mastication, convenience, and social interactions in patients with maxillectomy. 

## Figures and Tables

**Table 1:** Multiple comparisons of mean intelligibility scores across the stages of treatment.

Patients Groups	*n*	Preoperative SITS				Postoperative without Immediate Obturator 2 Day SITS				Postoperative with Obturator 2 Day SITS				Postoperative without Immediate Obturator 20 Day SITS				Postoperative with Immediate Obturator 20 Day SITS				Postoperative with Definitive Obturator SITS			
		Min	Max	Mean	SD	Min	Max	Mean	SD	Min	Max	Mean	SD	Min	Max	Mean	SD	Min	Max	Mean	SD	Min	Max	Mean	SD
**E** (Edentualism)	**10**	20	20	20	0.000	0	0	0.00	0.000	17	20	18.70	0.823	0	0	0.00	0.000	18	20	19	0.667	18	20	18.90	0.738
**PE** (Partial Edentualism)	**20**	20	20	20	0.000	0	1	0.05	0.224	16	20	18.75	1.251	0	1	0.05	0.224	18	20	19.35	0.671	19	20	19.40	0.503
**D** (Dentualism)	**11**	20	20	20	0.000	0	0	0.00	0.000	18	20	19.09	0.701	0	0	0.01	0.000	17	20	18.82	0.874	18	20	19.09	0.831
Mean Average SITS %	**41**	100%				0.02%				94.10%				0.02%				95.60%				95.97%			

***n*:** Number of Patients, **Min:** Minimum speech intelligibility test score (SITS), **Max:** Maximum SITS, **Mean:** Mean SITS, **SD:** Standard deviation.

**Table 2:** Test Statistics (Kruskal-Wallis Test and Grouping Variable: Group).

	Preoperative SITS	Post Operative without Obturator 2 day	Post Operative with Obturator 2 Day	Post Operative without Obturator 20 Day	Post Operative with Obturator 20 Day	Postoperative with Definitive Obturator
**Chi-Square (** *χ* ^2^ **)**	0.000	1.050	0.842	1.051	3.679	3.438
**df**	2	2	2	2	2	2
**Asymp. Sig. (** *p* **)**	1.000	0.592	0.656	0.593	0.159	0.179

**Table 3:** Statistical analysis of the speech intelligibility test scores in the different stages of treatment.

Patients Groups	Patients Groups	*t*	*p*
Preoperative SITS	Post-operative without immediate obturator 2 day SITS	819.000	<0.001
Preoperative SITS	Post-operative with immediate obturator 2 day SITS	7.333	<0.001
Preoperative SITS	Post-operative with immediate obturator 20 day SITS	7.515	<0.001
Preoperative SITS	Post-operative with definitive obturator SITS	7.591	<0.001
Post-operative without immediate obturator 2 day SITS	Post-operative with immediate obturator 2 day SITS	111.751	<0.001
Post-operative without immediate obturator 2 day SITS	Post-operative with immediate obturator 20 day SITS	152.826	<0.001
Post-operative without immediate obturator 2 day SITS	Post-operative with definitive obturator SITS	174.451	<0.001
Post-operative with immediate obturator 2 day SITS	Post-operative with immediate obturator 20 day SITS	2.080	0.044
Post-operative with immediate obturator 2 day SITS	Post-operative with definitive obturator SITS	2.639	0.012
Post-operative with immediate obturator 20 day SITS	Post-operative with definitive obturator SITS	0.723	0.474

## References

[1] Plank D.M., Weinberg B., Chalian V.A. (1981). Evaluation of speech following prosthetic obturation of surgically acquired maxillary defects. J. Prosthet. Dent..

[2] Wheeler R.L., Logemann J.A., Morton S.R. (1980). Maxillary reshaping prosthesis: Effectiveness in improving speech and swallowing of post-surgical oral cancer patients. J. Prosthet. Dent..

[3] Sawhney A., Dwivedi H., Singh S., Dhar S., Gupta S., Choudhary P. (2016). Hollow Bulb Obturator—A Simplified Approach. Arch. Dent. Med. Res..

[4] Suryakant C.D., Mantri S.S., Naitam D., Dube G., Gupta P., Dewangan A. (2013). A direct investment Method of Closed Two-piece hollow bulb obturator. Case Rep. Dent..

[5] McAndrews K.S., Rothenberger S., Minsley G.E. (1998). An innovative investment method for the fabrication of a closed hollow obturator prosthesis. J. Prosthet. Dent..

[6] Minsely G.E., Warren D.W., Hinton V. (1987). Physiologic Response to maxillary resection and subsequent obturation. J. Prosthet. Dent..

[7] Chalian V.A., Drane J.B., Standish S.M. (1971). Maxillofacial Prosthetics: Multidisciplinary Practice.

[8] Taylor T.D. (2000). Clinical Maxillofacial Prosthetics.

[9] Loh H.S., Tan P.H. (1989). Prosthodontic management of maxillofacial defects after cancer surgery. Singap. Med. J..

[10] Gowda M.E., Mohan M.S., Verma K., Roy I.D. (2013). Implant rehabilitation of partial maxillectomy edentulous patient. Contemp. Clin. Dent..

[11] Brown K.E. (1969). Complete Denture Treatment in patients with resected mandibles. J. Prosthet. Dent..

[12] Swoope C.C. (1969). Prosthetic Management of the resected edentulous mandibles. J. Prosthet. Dent..

[13] Scaaf N.G. (1976). Oral construction for edentulous patients after partial mandibulectomies. J. Prosthet. Dent..

[14] Rahn A.O. (1970). Maxillofacial Prosthetics Principles and Concepts.

[15] Patton D.W., Ali A., Davies R., Fardy M.J. (1994). Oral rehabilitation and quality of life following the treatment of oral cancer. Dent. Update.

[16] Usui H. (1994). Evaluation of maxillary prosthesis for better QOL, Nihon. Jibiinkoka Gakkai Kaiho.

[17] Bedard J.F., Toljanic J.A. (2010). Management of Large Maxillary Defects Following Tumor Resection: Prosthetic and Prosthodontic Considerations. www.uptodate.com/online/content/topic.do?topicKey=head_can/13669.

[18] Curtis T.A., Beumer J., Beumer J., Curtis T.A., Marunick M.T. (1996). Maxillofacial rehabilitation: Prosthodontic and surgical considerations. Restoration of Acquired Hard Palate Defects: Etiology, Disability, and Rehabilitation.

[19] Won-suck O.H., Roumanas E. (2006). Alternate technique for fabrication of a custom impression tray for definitive obturator construction. J. Prosthet. Dent..

[20] Keyf F. (2001). Obturator prosthesis for hemimaxillectomy patients. J. Oral Rehabil..

[21] Wolfaardt J.F. (1989). Modifying a surgical obturator prosthesis into an interim obturator. A Clinical report. J. Prosthet. Dent..

[22] Rieger J., Wolfaardt J., Seikaly H., Jha N. (2002). Speech outcomes in patients rehabilitated with maxillary obturator prosthesis after maxillectomy: A prospective study. Int. J. Prosthodont..

[23] Sullivan M., Gaebleer C., Beukelman D., Mahanna G., Marshall J., Lydiatt D., Lydiatt W. (2002). Impact of palatal prosthodontic intervention on communication performance of patients maxillectomy defects: A multilevel outcome study. Head Neck.

[24] Rathee M., Bhoria M., Malik P. (2014). Prosthodontic rehabilitative therapy through surgical obturator for maxillectomy patient: A Review. Cancers Rev..

[25] Aramany M.A. (2001). Basic principles of obturator design for partially edentulous patients. Part II: Design principles. J. Prosthet. Dent..

[26] Aramany M.A. (1978). Basic principles of obturator design for partially edentulous patients. Part I: Classification. J. Prosthet. Dent..

[27] Tanaka Y., Gold H.O., Pruzansky S. (1977). A simplified technique for fabricating a lightweight obturator. J. Prosthet. Dent..

[28] Wu L.Y., Schaaf N.G. (1989). Comparison of weight reduction in different designs of solid and hollow obturator prostheses. J. Prosthet. Dent..

[29] Mehta S., Mascarenhas E. (2014). Closed hollow obturator—An elixir to the cancer patients. Int. J. Sci. Stud..

[30] Adisman I.K. (1990). Prosthesis serviceability for acquired jaw defects. Dent. Clin. N. Am..

[31] Aramany M.A., Drane J.B. (1972). Effect of nasal extension sections on the voice quality of acquired cleft palate patients. J. Prosthet. Dent..

[32] Oral K., Aramany M.A., McWilliams B.J. (1979). Speech intelligibility with the buccal flange obturator. J. Prosthet. Dent..

[33] Suha T., Timucin B., Asim A.M., Mustafa O.M. (2009). Articulation performance of patients wearing obturators with different buccal extension designs. Eur. J. Dent..

[34] Jain M., Bulbule N., Anasane N. (2017). A collaborative approach towards speech analysis in patients rehabilitated with maxillary obturator prosthesis: Case review. Int. J. Curr. Res..

[35] Bohle G., Rieger J., Huryn J., Verbel D., Hwang F., Zlotolow I. (2005). Efficacy of speech aid prostheses for acquired defects of the soft palate and velopharyngeal inadequacy-clinical assessments and cephalometric analysis: A Memorial Sloan-Kettering Study. Head Neck.

[36] Jacob R.F. (2000). Clinical Management of the Edentulous Maxillectomy Patient in Clinical Maxillofacial Prosthetics.

[37] Umino S., Masuda G., Ono S., Fujita K. (1998). Speech intelligibility following maxillectomy with and without prosthesis: An analysis of 54 cases. J. Oral Rehabil..

[38] Arigbede A.O., Dosumu O.O., Shaba O.P., Esan T.A. (2006). Evaluation of Speech in Patients with Partial Surgically Acquired Defects: Pre and Post Prosthetic Obturation. J. Contemp. Dent. Pract..

[39] Fenn H.R.B., McGregor A.R. (1989). Fenn, Liddelow F and Gimson’s Clinical Dental Prosthetics.

[40] Blair F.M., Hunter N.R. (1998). The hollow box maxillary obturator. Br. Dent. J..

[41] Lawson M.A. (1968). Speech and its relation to dentistry: The influence of oral structures on speech. Dent. Pract. Dent. Rec..

[42] Kipfmueller L.J., Lang B.R. (1972). Presurgical maxillary prosthesis: An analysis of speech intelligibility. J. Prosthet. Dent..

[43] Carvalho-Teles V.D., Pegoraro-Krook M.I., Lauris J.R.P. (2006). Speech evaluation with and without palatal obturator in patients submitted to maxillectomy. J. Appl. Oral Sci..

[44] Bloomer H.H., Hawk A.W. (1973). Orofacial Anomalies: Clinical and Research Implications. Speech Considerations: Speech Disorders Associated with Ablative Approaches to Learning.

[45] Majid A.A., Weinberg B., Chalian V.A. (1974). Speech ineligibility following prosthetic obturation of surgically acquired maxillary defects. J. Prosthet. Dent..

[46] Yoshida H., Michi K., Ohawa T. (1990). Prosthetic treatment for speech disorders due to surgically acquired maxillary defects. J. Oral Rehabil..

[47] Watson R.M. (1985). Assessing effective obturation. J. Prosthet. Dent..

[48] Fletcher S.G., Jacob R.F., Kelly D.H., Krieger B.M., Martin D.E., Myers E.N., Rolnick M.I. (1991). Speech production in rehabilitation of head and neck cancer patients. Head Neck.

[49] Rieger J.M., Wolfaardt J.F., Jha N., Seikaly H. (2003). Maxillary obturators: The relationship between patient satisfaction and speech outcome. Head Neck.

[50] Kornblith A.B., Zlotolow I.M., Gooen J., Huryn J.M., Lerner T., Strong E.W., Shah J.P., Spiro R.H., Holland J.C. (1996). Quality of life of maxillectomy patients using an obturator prosthesis. Head Neck.

[51] Pegoraro-Krook M.I. (1995). Avaliação dos Resultados de Fala de Pacientes que Apresentam Inadequação Velofaríngea e que Utilizam Prótese de Palato. Tese de Doutorado.

[52] Yoshida H., Furuya Y., Shimodaira K., Kanazawa T., Kataoka R., Takahashi K. (2000). Spectral characteristics of hypernasality in maxillectomy patients. J. Oral Rehabil..

